# Marine-Derived Quorum-Sensing Inhibitory Activities Enhance the Antibacterial Efficacy of Tobramycin against *Pseudomonas aeruginosa*

**DOI:** 10.3390/md13010001

**Published:** 2014-12-24

**Authors:** Alessandro Busetti, George Shaw, Julianne Megaw, Sean P. Gorman, Christine A. Maggs, Brendan F. Gilmore

**Affiliations:** 1 School of Pharmacy, Queen’s University Belfast, 97 Lisburn Rd, Belfast BT9 7BL, UK; E-Mails: a.busetti@qub.ac.uk (A.B.); gshaw08@qub.ac.uk (G.S.); j.megaw@qub.ac.uk (J.M.); s.gorman@qub.ac.uk (S.P.G.); 2 School of Biological Sciences, Medical Biology Center, 97 Lisburn Road, Belfast BT9 7BL, UK; E-Mail: c.maggs@qub.ac.uk

**Keywords:** antibiotic resistance, antimicrobial synergy, quorum sensing inhibitors, marine bacteria, biofilm, marine biodiscovery, tobramycin, *Pseudomonas aeruginosa*

## Abstract

Bacterial epiphytes isolated from marine eukaryotes were screened for the production of quorum sensing inhibitory compounds (QSIs). Marine isolate KS8, identified as a *Pseudoalteromonas* sp., was found to display strong quorum sensing inhibitory (QSI) activity against acyl homoserine lactone (AHL)-based reporter strains *Chromobacterium violaceum* ATCC 12472 and CV026. KS8 supernatant significantly reduced biofilm biomass during biofilm formation (−63%) and in pre-established, mature *P. aeruginosa* PAO1 biofilms (−33%). KS8 supernatant also caused a 0.97-log reduction (−89%) and a 2-log reduction (−99%) in PAO1 biofilm viable counts in the biofilm formation assay and the biofilm eradication assay respectively. The crude organic extract of KS8 had a minimum inhibitory concentration (MIC) of 2 mg/mL against PAO1 but no minimum bactericidal concentration (MBC) was observed over the concentration range tested (MBC > 16 mg/mL). Sub-MIC concentrations (1 mg/mL) of KS8 crude organic extract significantly reduced the quorum sensing (QS)-dependent production of both pyoverdin and pyocyanin in *P. aeruginosa* PAO1 without affecting growth. A combinatorial approach using tobramycin and the crude organic extract at 1 mg/mL against planktonic *P. aeruginosa* PAO1 was found to increase the efficacy of tobramycin ten-fold, decreasing the MIC from 0.75 to 0.075 µg/mL. These data support the validity of approaches combining conventional antibiotic therapy with non-antibiotic compounds to improve the efficacy of current treatments.

## 1. Introduction

The emergence, spread and evolution of antimicrobial resistance (AMR) amongst microorganisms is an ancient biological phenomenon and a direct consequence of the selective pressure imposed by natural selection [1]. The mechanisms that lead to the emergence and diffusion of AMR are multiple and include mutation (stochastic or mutagen-induced), gradual increased tolerance to sub-lethal concentrations of compounds, horizontal gene transfer through mobile genetic elements (plasmids, transposons and insertion sequences) and recombination events (such as those between a bacteriophage and a bacterial host) [2,3].

The discovery of antibiotics [4] and their use as therapeutics has resulted in drastic decreases in morbidity and mortality associated with bacterial infectious diseases (the “antibiotic era”). Paradoxically, anthropogenic use of antibiotics both in the clinical setting and in non-therapeutic theatres of human activity such as agriculture and zootechny [5] has accentuated natural selective pressure on bacterial pathogens, accelerating the acquisition and diffusion of resistance, undermining the efficacy of antibiotics in the clinical context. For example, MRSA infections in the US are currently responsible for more deaths than HIV/AIDS and tuberculosis combined [6,7]. The selective pressures induced by antimicrobial use are particularly evident in the nosocomial environment where clear relationships between antimicrobial use and the emergence of multiresistant, extensively drug-resistant and pan-drug-resistant strains have been observed [8,9]. Despite concerted efforts, the discovery of new antibiotics has considerably slowed down and the number of new antibacterial drugs gaining FDA approval in the United States continues to fall [10]. Without such tools we can expect to witness a substantial rise in incurable chronic infection and fatality in both underdeveloped and developed regions of the globe. This imminent global health threat has spurred research investigating novel therapeutic strategies based on new targets and approaches aimed at circumventing the emergence of antimicrobial resistance.

The discovery and elucidation of the mechanisms underlying bacterial density-dependent cell-to-cell signaling relying on small diffusible signaling molecules, known as quorum sensing (QS) [11], has offered promising novel targets for antivirulence approaches aimed at disarming rather than killing human pathogens during the course of colonization and infection. In fact, many Gram positive and Gram negative bacteria use QS to coordinate behaviors and regulate a diverse array of physiological activities including swarming [12], bioluminescence [13], symbiosis [14], virulence [15], competence [16], production of extracellular enzymes [17], conjugation [18], sporulation [19], antibiotic synthesis [20], biofilm formation [21,22] and to regulate the production of factors used to scavenge for nutrients, actuate immune suppression, provide scaffolding for biofilms to grow and aid motility [23].

The dramatically rapid and continuous emergence of antibiotic resistance in the clinical context necessitates the urgent identification of novel strategies for treating bacterial infections, including non-antibiotic based approaches. QS inhibition represents an emerging, promising strategy to render pathogens more susceptible to antibiotics and hinder adaptation to host immune response, attenuating bacterial virulence by limiting the emergence of pathogenic traits [15,24]. In fact, it has been shown that by inhibiting QS, a disruption of the signaling systems controlling the production and release of a number of virulence factors is achieved. For example, the expression of AHL lactonase (enzymes which catalyze the opening of the lactone ring component of the AHL molecule [24]) in the human pathogens *P. aeruginosa* and *Burkholderia* species resulted in large decreases in virulence gene expression and swarming motility [25] and the attenuation of virulence [26,27]. In agricultural applications, the expression of AHL lactonase in transgenic tobacco leaves and potatoes significantly enhanced resistance against *Erwinia carotovora* infections, a pathogen reliant on AHL-mediated quorum-sensing for its virulence [28]. Halogenated furanones are another well-characterized class of marine-derived compounds shown to possess QSI activity against a broad range of bacteria [29,30]. These compounds antagonize AHL-dependent gene expression through accelerated degradation of the LuxR-type transcriptional activator [31], inhibiting virulence factor expression [15]. Overall, QS inhibition can be considered an ‘anti-virulence’ approach based on the use of molecules capable of disarming pathogens within their host by targeting specific factors necessary for successful infection, such as toxin biosynthesis and function, toxin delivery, virulence gene regulation, or cell adhesion [32,33,34].

Although the role of QS in regulating antimicrobial susceptibility still needs to be fully elucidated, several studies using both *in vitro* and *in vivo* models have shown increased susceptibility of pathogenic bacteria to antibiotics when used in combination with QSIs [35,36,37]. These promising results suggest the possibility of using QSIs in combination with current and future antibiotics at lower therapeutic concentrations, reducing toxicity for the patient. Moreover, as QS is not involved in essential cellular processes, QSI strategies are less likely to generate resistance [24,38]. It has been hypothesized that the emergence of QSI resistance would be selected *in vivo* during infection whenever QS promotes colonization, systemic spread, or immune evasion [39]. However the use of a joint antimicrobial/antivirulence approach would considerably reduce the likelihood of the emergence of such a resistance.

*Pseudomonas aeruginosa* is a common Gram negative opportunistic pathogen associated with biofilm-related nosocomial infections [40,41] and to chronic lung infection in cystic fibrosis (CF) sufferers, where such infections constitute the major cause of patient morbidity and mortality [42,43]. It utilizes three different QS circuits to regulate the production of virulence factors and promote biofilm maturation [44,45]. The lasI/lasR and rhlI/rhlR QS circuits are based on AHL signaling molecules and regulate 170–400 genes via a complex network [30,46,47] making this pathogen an excellent candidate for the use of antivirulence approaches based on the use of QSIs.

The aqueous nature of the marine environment allows the constant development of a ubiquitous microbial biofilm covering the majority of submerged surfaces [48]. In this environment, natural selection has bestowed an evolutionary advantage to microorganisms with an array of chemical defenses enabling them to compete for space, nutrients and light during the colonization of biotic or abiotic surfaces. In particular, a large number of marine natural compounds isolated from a variety of marine organisms possess QSI activity [49] suggesting QS inhibition has evolved as a natural, widespread, antimicrobial strategy with significant impact on biofilm formation, making the marine ecosystems an ideal source for the discovery of QS inhibitors with the potential to increase susceptibility of human pathogens to antibiotics.

## 2. Results and Discussion

**Figure 1:**
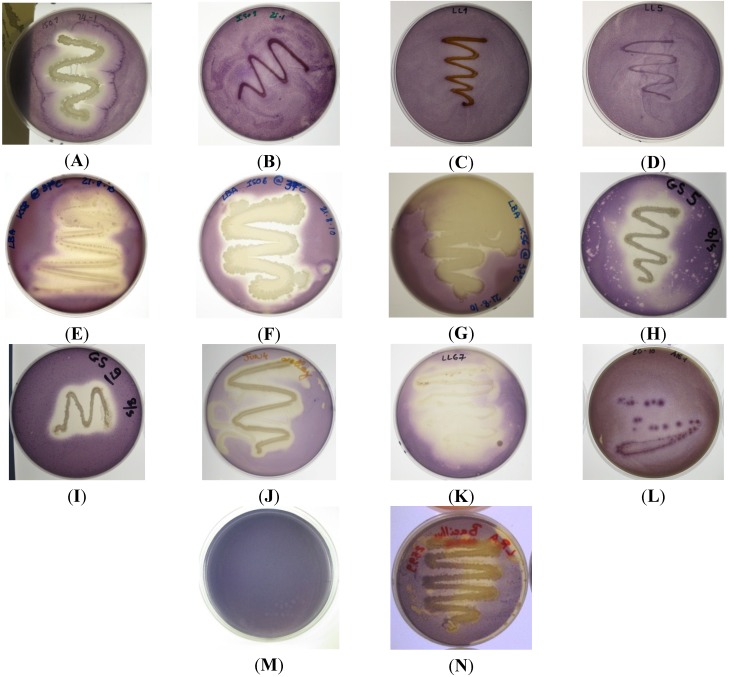
Quorum sensing inhibitory compound (QSI) screening of bacterial isolates using reporter strain *C. violaceum* ATCC 12472; (**A**) ISO1, weak antimicrobial activity, strong QSI activity; (**B**) ISO9 no QSI activity detected; (**C**) LL1 no QSI activity detected; (**D**) LL5 no QSI activity detected; (**E**) KS8 strong QSI activity; (**F**) ISO6 strong QSI activity; (**G**) KS6 strong QSI activity; (**H**) GS5 medium-strong QSI activity and weak antimicrobial activity; (**I**) GS9 no QSI activity and medium-strong antimicrobial activity; (**J**) JUN4 strong QSI activity; (**K**) LL67 strong QSI activity; (**L**) A15-1 weak QSI activity; (**M**) *C. violaceum* ATCC 12472, negative QSI control; (**N**) *B. cereus* NCTC 9945 harboring the aiiA lactonase gene (positive QSI control) displaying modest QSI activity.

Initially, 26 marine bacterial isolates were screened for the production of QSIs against the Gram negative AHL-based reporter strains *C. violaceum* ATCC 12472, and CV026. Nine isolates (ISO1, ISO2, ISO6, KS6, KS8, GS5, JUN4, LL67 and A15-1) were found to strongly inhibit QS-dependent violacein production in both *C. violaceum* ATCC 12472 (Figure 1) and *C. violaceum* CV026 (data not shown). *C. violaceum* ATCC 12472 regulates pigment production in response to *N*-hexanoyl-l-homoserine lactone (C6-HSL) [50]. Although reporter strain *C. violaceum* ATCC 12472 is a useful tool to detect QSI activity, it provides no further information with regards to the mechanism of action of the compounds involved in the QSI activity displayed by isolates. The three key targets of a QSI drug approach against Gram negative pathogens that use an AHL-based QS system are the signal molecule generator (LuxI-type synthases), the signal molecule itself (AHL) and the signal molecule receptor (Lux-R-type transcription factors. For example, it is known that an inactivation of the LuxI-type synthase can block the production of the relative AHL signaling molecule [24]. QS can also be inhibited by targeting the signal molecule itself with the main proposed strategies being metabolic, chemical and enzymatic degradation or inactivation. The third target for QSI is the LuxR transcription factor. For example, small AHL analogues have been used to block the activation of LuxR inhibiting downstream activation of QS-regulated pathways [24,51]. *C. violaceum* (CV026) is a mini-Tn5 mutant where the transposon insertion sites have been mapped to a putative repressor locus and to a *lux*I homologue (*cvi*I) and in which violacein production can be restored by incubation with supernatants from the wild-type strain [52,53]. As the QSI assay using reporter strain CV026 requires exogenous addition of C6-AHL signaling molecule, the results suggest the QSI activity of KS8 observed against the wild type *C. violaceum* is not attributable to the inactivation of the components involved in the synthesis of the signal molecule. Based on the strength of the QSI activity and the lack of antimicrobial activity displayed by isolate KS8 against reporter strains *C. violaceum* ATCC 12472 and CV026, screening of this isolate for QSI was repeated using reporter strain *Serratia* sp. ATCC 39006. This reporter strain produces the red pigment prodigiosin in response to the short-chain signal *N*-butanoyl-l-homoserine lactone (C4-HSL) [54], the same AHL used in the *Pseudomonas aeruginosa* rhlI/rhlR QS system which regulates the expression of *rhlAB* (rhamnolipid), *lasB*, *aprA*, RpoS, cyanide, pyocyanin [55,56,57] and the lectins PA-I and PA-II [58]. Isolate KS8 caused inhibition of pigment production in its proximity, indicative of putative QSI activity (Supplementary Figure S1). When interpreted in conjunction to the results on *C. violaceum* ATCC 12472 and CV026, the QSI activity observed against *Serratia* sp. ATCC 39006 suggests the inhibition of QS by KS8 is likely to be broad-spectrum rather than dependent on the structure of the AHL molecule itself.

**Figure 2:**
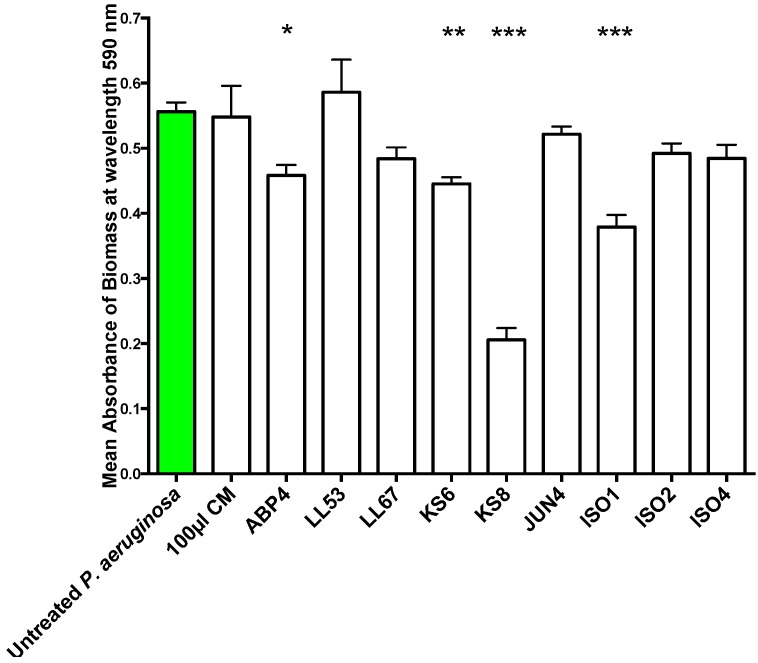
Biofilm prevention assay. 24 h biofilm biomass (mean absorbance of biomass at 590 nm) of *P. aeruginosa* PAO1 (green) following 24 h exposure to 100 μL of sterile 3-day supernatants of marine isolates ABP4, LL53, LL67, KS6, KS8, JUN4, ISO1, ISO2, ISO4. CM = Conditioned media. Differences in mean absorbance were compared to the untreated control at each time-point and considered significant when *p* < 0.05 (* *p* < 0.05, ** *p* < 0.01, *** *p* < 0.001) according to the non-parametric Kruskal Wallis test with Dunn’s Multiple Comparison Test.

**Figure 3:**
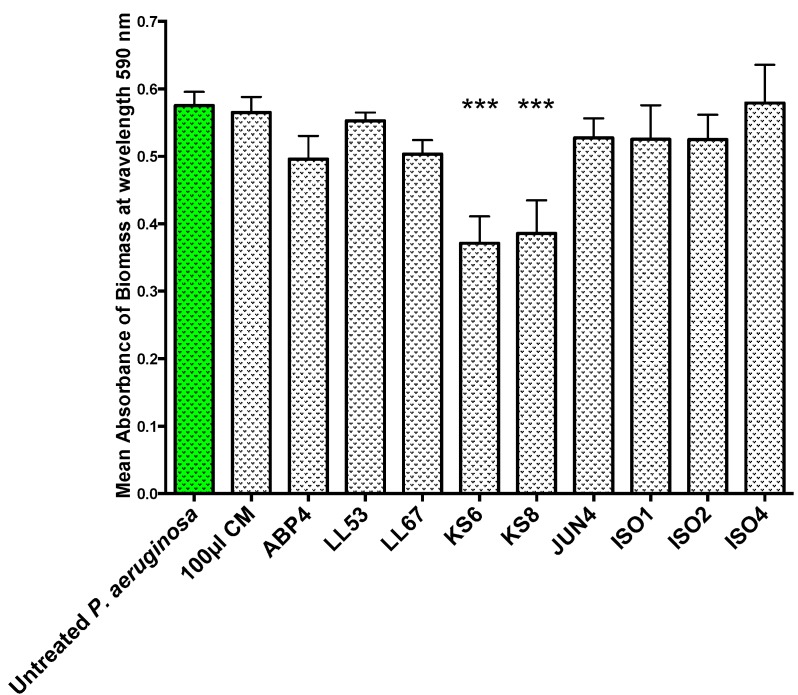
Biofilm removal assay. 48 h biofilm biomass (mean absorbance of biomass at 590 nm) of *P. aeruginosa* (PAO1) biofilms following 24 h challenge using 100 μL of sterile 3-day supernatants of marine isolates ABP4, LL53, LL67, KS6, KS8, JUN4, ISO1, ISO2, ISO4. CM = Conditioned media. Differences in mean absorbance were compared to the untreated control at each time-point and considered significant when *p* < 0.05 (* *p* < 0.05, ** *p* < 0.01, *** *p* < 0.001) according to the non-parametric Kruskal Wallis test with Dunn’s Multiple Comparison Test.

To test whether the QSI activity displayed by the nine bacterial isolates was correlated to antibiofilm activity, the isolates’ filter-sterilized supernatants were screened for the ability to inhibit biofilm formation (Figure 2) and eradicate pre-established biofilms (Figure 3) of *P. aeruginosa* PAO1. The supernatant of isolate KS8 caused a significant reduction in the mean absorbance of stained biomass of *P. aeruginosa* PAO1 biofilm (−63%) (Figure 2) over 24 h (Figure 2 and Table 1), suggesting the presence of compounds capable of interfering with the maturation of the biofilm. For example, the presence of growth-inhibiting bioactives could be responsible for slowing down biofilm growth, whilst failing to have an effect on the long-term final total biomass produced by the test pathogen

KS8 supernatant also caused a significant reduction of *P. aeruginosa* PAO1 (−33%) biomass in pre-established 24 h biofilms following 24 h treatment with KS8 supernatant (biofilm removal) (Figure 3, Table 1). The mean absorbance of biofilm biomass for 24 h PAO1 biofilms (0.67 O.D. at 590 nm) did not vary significantly from the mean absorbance of untreated 48 h biofilms confirming biofilms had reached full maturity and remained relatively intact during the 24 h test period. The results of the biofilm eradication assay also suggest the inhibition of biofilm formation is unlikely to be attributable to growth inhibition, and although possibly multifactorial in nature, the reduction in biofilm biomass could involve the upregulation of dispersal mechanisms. In fact, compounds such as dispersin B and nitric oxide have been shown to affect biofilm formation by promoting cell dispersal [59].

All of the nine isolates tested failed to completely prevent biofilm formation or completely eradicate pre-established test biofilms. Several concurrent factors could have contributed to the modest antibiofilm activities observed. Firstly, as the isolates being tested produced putative QSIs, it is unlikely for QS inhibition to completely prevent adhesion or promote complete biofilm dispersion/eradication. In fact, QSIs are rather more likely to affect the structure, physiology and maturation of the biofilm. Moreover, to avoid stressing the test biofilms due to starvation, supernatants were diluted in fresh Luria Bertani (LB) broth and as a result, the bioactives present could have been diluted below effective concentrations.

**Table 1:** Summary of the percent reduction of *P. aeruginosa* (PAO1) biofilm biomass following 24 h challenge using sterile 72 h KS8 supernatant during biofilm formation and on mature, pre-established 24 h *P. aeruginosa* (PAO1) biofilms (biofilm removal assay). Differences in mean absorbance compared to the untreated control of each time-point were considered significant when *p* < 0.05 (* *p* < 0.05, ** *p* < 0.01, *** *p* < 0.001) according to the non-parametric Kruskal Wallis test with Dunn’s Multiple Comparison Test.

Isolate	Closest Typed Species (NCBI)	Biofilm Formation	Biofilm Removal
		*PAO1*	*PAO1*
KS8	*Pseudoalteromonas* sp.	−63% (***)	−33% (***)

The antibiofilm activity displayed by the culture supernatant of isolate KS8 appeared to be more significant during biofilm formation than in the removal of pre-established 24 h biofilms. There are several factors that could have contributed to this result. The copious production of extracellular polymeric substances, a characteristic feature of established and mature *P. aeruginosa* biofilms, can limit the diffusion of bioactives and is often the site of enzymatic deactivation/degradation of antimicrobials providing protection against mechanical removal, predation, and exogenous antimicrobial insult whilst securing the presence of a suitable biofilm microenvironment [60,61].

Based on the consistent antibiofilm activity displayed in the microtitre crystal violet assay by the supernatant of isolate KS8, both preventing biofilm formation and promoting the eradication of mature 24 h biofilms of *P. aeruginosa* PAO1, this isolate was selected for further studies. A 758 bp 16S rRNA gene sequence was obtained following DNA extraction and PCR and used to identify KS8 (isolated from the marine Bryozoan *Scrupocellaria* sp.) using the basic local alignment search tool (BLAST) of the NCBI database. Five accession numbers belonging to the genus *Pseudoalteromonas* were found to share a 99% sequence identity with KS8, all with an E-value of zero and a max score of 1393 allowing identification to the genus level. Various members of the genus *Pseudoalteromonas* have been shown to produce antibiofilm compounds. For example, the exoproducts of* Pseudoalteromonas* sp. 3J6 isolated from marine biofilms grown on glass slides impaired biofilm formation of *Paracoccus* sp. 4M6, *Vibrio* sp. D01 and the three human-pathogenic species *Pseudomonas aeruginosa*, *Salmonella enterica*, and *Escherichia coli*, leading to a higher percentage of non-viable cells in 48 h biofilms and reduced bacterial attachment on coated glass slides [62]. Another member of this genus, *Pseudoalteromonas tunicata* D2, has been shown to secrete the autotoxic 190 kDa dimeric antibacterial protein AlpP which gives a competitive advantage for the latter for biofilm formation in the marine environment and for the colonization of the surface of the green alga *Ulva australis* [62]. In fact, AlpP is able to inhibit a variety of marine and medically important bacteria [63] and has been shown to be particularly autotoxic with an MIC of 4 μg/mL [64]. It has been hypothesized that by working through an apoptosis-like mechanism, the autoxicity of AlpP provides a dispersal mechanism to biofilm dwelling *P. tunicata* [65], where the self-induced lysis of cells causes sections of the biofilm to rip and shed but also supplies nutrients to survivor cells resistant to AlpP [62]. The survivor cells contained in the “floccules” of shed biofilm actively disperse in the surrounding water in search of new surfaces to colonize [66,67]. The genus *Pseudoalteromonas* is also well known for the production of antibacterial, antifungal, antiviral and/or algicidal molecules. For example, a closely related species, *Pseudoalteromonas atlantica*, isolated from *Cancer pagurus* (edible crab) [68] was found to produce a variety of biologically active extracellular compounds such as extracellular polysaccharides responsible for influencing succession in marine communities, eventually leading to the settlement of higher organisms such as tunicates and oyster larvae [69].

**Figure 4:**
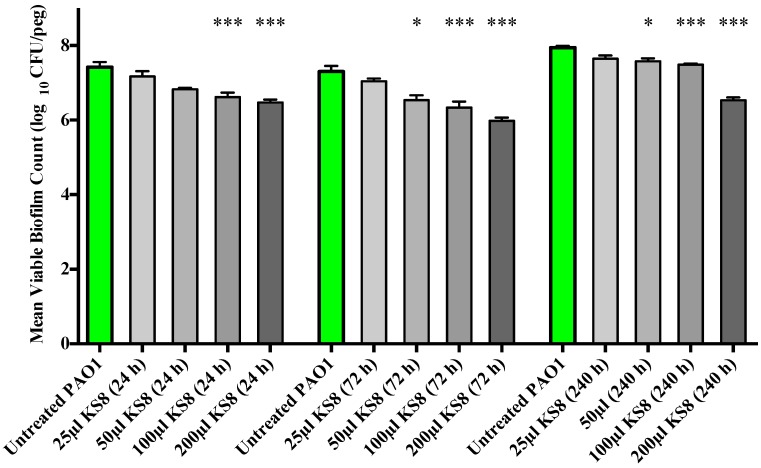
Prevention of biofilm formation by isolate KS8 supernatant and determination of the optimal incubation period for bioactive production. *P. aeruginosa* (PAO1) mean biofilm counts following 24 h exposure to sterile 1-day, 3-day and 10-day supernatants of isolate KS8. PAO1 biofilms were grown on the Calgary Biofilm Device for 24 h in LB broth at 37 °C and 150 rpm. Differences in mean absorbance were compared to the untreated control at each time-point and considered significant when *p* < 0.05 (* *p* < 0.05, ** *p* < 0.01, *** *p* < 0.001) according to the non-parametric Kruskal Wallis test with Dunn’s Multiple Comparison Test.

**Figure 5:**
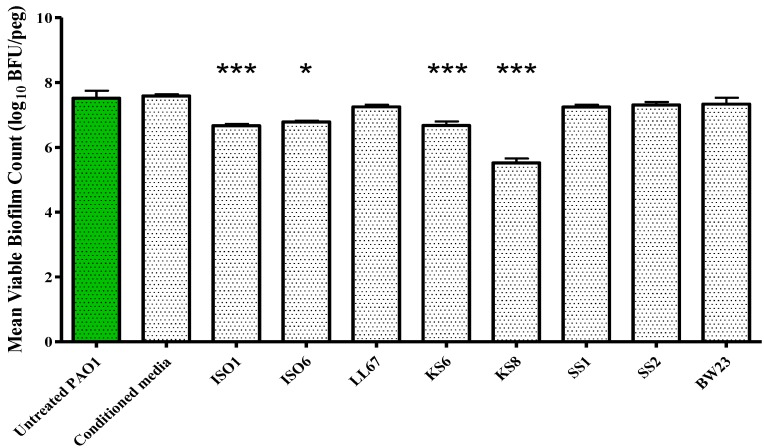
Biofilm eradication assay. *P. aeruginosa* PAO1 biofilms were grown on the Calgary Biofilm Device for 24 h in LB broth at 37 °C and 150 rpm prior to challenge. Mean viable biofilm counts obtained following 24 h exposure to 100 μL of sterile 3-day supernatant of isolates ISO1, ISO6, LL67, KS6, KS8, SS1, SS2, and BW23. Each value is expressed as the mean and standard deviation of at least six replicates. Differences in mean absorbance were compared to the untreated control at each time-point and considered significant when *p* < 0.05 (* *p* < 0.05, ** *p* < 0.01, *** *p* < 0.001) according to the non-parametric Kruskal Wallis test with Dunn’s Multiple Comparison Test.

As the crystal violet microtitre plate assay measures biomass but fails to take into account cell viability, the study of the prevention of biofilm formation and biofilm eradication following exposure to the bioactives produced by isolate KS8 was conducted using the Calgary Biofilm Device (CBD, MBEC™ assay for Physiology & Genetics, Innovotech, Edmonton, Alberta, Canada). To determine an optimal incubation period for the production of bioactives, biofilm formation studies were conducted using 24, 72 and 240 h supernatants. Biofilm formation of *P. aeruginosa* PAO1 was significantly inhibited by the higher concentrations (100 and 200 μL) of all sterile KS8 supernatants (Figure 4) however the 72 h supernatant also caused a significant reduction when diluted in a 1:4 ratio (50 μL), and was thus chosen as the incubation time for the studies to follow. Although significant, the mechanism of such inhibition remains to be elucidated and it is unclear whether the reduction in biofilm formation by *P. aeruginosa* PAO1 is attributable to a reduced capacity to metabolize media, an increased rate of dispersal, a growth inhibitory effect or to a combination of effects. The sterile 3-day supernatant of isolate KS8 also displayed a significant antibiofilm activity (biofilm eradication) against mature *P. aeruginosa* PAO1, causing a 99% reduction in biofilm viable counts (Figure 5 and Table 2). The viable counts (log_10 _bfu/peg) of untreated 24 h PAO1 biofilms (7.264) did not vary significantly from the viable counts of untreated 48 h PAO1 biofilms (7.444) confirming the activities observed did not depend on the removal of biofilm or on the inhibition of growth during the test period (Supplementary Figure S2). Further work will be necessary to elucidate the mechanisms underlying the inhibition of biofilm formation and the biofilm eradication observed against this pathogen.

**Figure 6:**
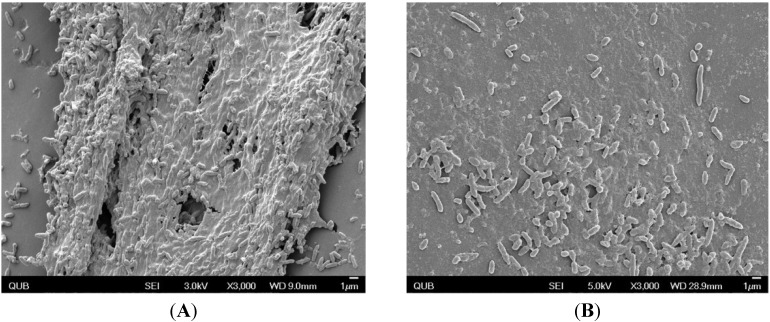
Scanning electron micrographs of (**A**) *P. aeruginosa* PAO1 biofilms grown on the CBD for 24 h at 37 °C; (**B**) PAO1 biofilms treated with KS8 8-day supernatant. Pictures were taken using using a field emission scanning electron microscope FE-SEM (JEOL JSM-6500F FE-SEM, JEOL Ltd., Tokyo, Japan).

The effect of KS8 supernatant on pre-established *Pseudomonas aeruginosa* PAO1 biofilms was examined by scanning electron microscopy (SEM) with the primary purpose of investigating the effect on cell integrity and the resulting morphological changes experienced by constituent cells, as well as effects on biofilm architecture as a whole. PAO1 biofilms treated with KS8 supernatant appeared to confirm the reduction in biomass absorbance and viable counts lacking the presence of copious exoploymeric matrix and the absence of a confluent lawn of biofilm growth (Figure 6).

To optimize the production of bioactives prior to solvent extraction of culture supernatants, the production of putative QSIs was determined across a range of temperatures and incubation times. The optimal incubation temperature for bioactive production was found to be 37 °C and the optimal cultivation time was found to be 3–10 days depending on the pathogen being challenged (Supplementary Figures S3 and S4). Disc diffusion assays using sterile supernatant failed to identify the presence of antibiotic activity against *P. aeruginosa* PAO1 suggesting the antibiofilm activity observed is not attributable to a bactericidal effect.

**Table 2:** Biofilm eradication assay. Log_10_ bfu/peg biofilm viable counts and percent reduction of pre-established *P. aeruginosa* (PAO1) biofilms challenged with 100 μL of 3-day supernatant of marine isolate KS8 in the CBD (24 h at 37 °C and 95% relative humidity at 150 rpm). Differences in log_10_(bfu/peg) were considered significant when *p* < 0.05 (* *p* < 0.05, ** *p* < 0.01, *** *p* < 0.001) according to the non-parametric Kruskal Wallis test with Dunn’s Multiple Comparison Test. Values for the percent reduction in biomass absorbance are also included.

	Biofilm Formation		Biofilm Eradication	
	Log_10_ bfu/peg	Δ* (*Log10 bfu/peg) and % Reduction	Log_10_ bfu/peg	Δ* (*Log10 bfu/peg) and % Reduction
**Untreated PAO1**	7.30		7.52	
**KS8**	6.34	−0.96	5.52	−2.00 (***)
		(−89.04%)		(−99.00%)

**Figure 7:**
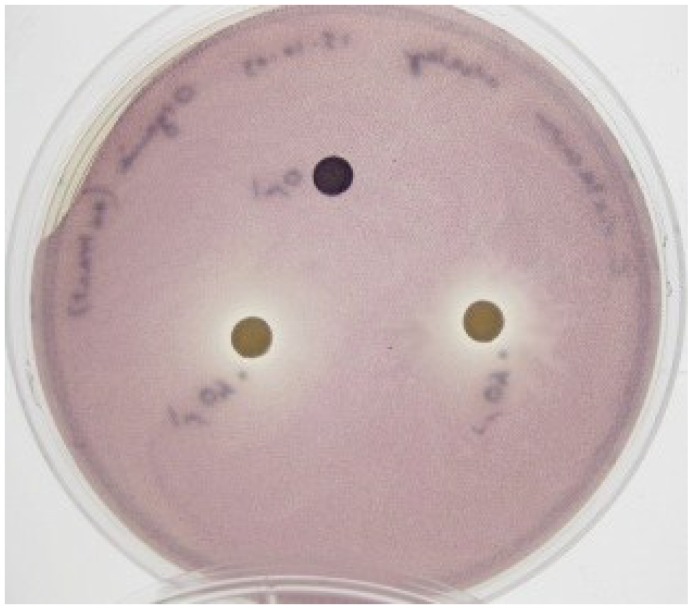
Disc diffusion assays on QSI-reporter strain *C. violaceum* ATCC 12472 using the crude organic extract of marine isolate KS8. A disc loaded with 60 μL of EtOAc and allowed to air-dry was included in the plate as a solvent control (top). Two discs were loaded with 40 μL of the crude organic extract of marine isolate KS8 (10 mg/mL). QSI activity was detected as an opaque halo surrounding the test discs.

Solvent extraction was performed in an attempt to extract and concentrate the antibiofilm compounds produced by marine isolate KS8. Ethyl acetate extraction of supernatants is a standard technique routinely employed for the initial crude extraction of bioactives from microorganisms [70,71]. Growth curves of planktonic *P. aeruginosa* PAO1 treated with KS8 crude extract excluded growth inhibitory effects or antimicrobial effects at concentrations of the extract up to 1 mg/mL (Supplementary Figure S5). Moreover, screening of the organic crude extract of isolate KS8 against *C. violaceum* ATCC 12472 (Figure 7) and *Serratia sp.* ATCC 39006 using the disc diffusion assay confirmed the successful extraction of compounds with putative QSI activity. Heating the crude extract at 60 °C for 30 min did not affect the QSI activity displayed in the disc diffusion assay using *C. violaceum* ATCC 12472 (data not shown) suggesting the bioactive compound(s) involved are unlikely to be proteinaceous.

KS8 crude organic extract was tested for antimicrobial activity against a panel of Gram negative and Gram positive pathogens (Table 3). The crude organic extract of isolate KS8 displayed a selective antimicrobial activity against Gram negative pathogens inhibiting the growth of *P. aeruginosa* PAO1 (MIC = 2 mg/mL) and displaying a bactericidal activity against* E. coli* ATCC 11303 (MIC = 0.5 mg/mL, MBC = 4 mg/mL) and P*. mirabilis* ATCC 7002 (MIC = 1 mg/mL, MBC = 2 mg/mL). Although the MBEC value of the crude extract of KS8 was found to be greater than 4 mg/mL, a 2-log reduction (99%) was observed against mature pre-established PAO1 biofilms.

The contribution of QS controlled factors (including toxA, lasA, lasB, rhamnolipids and pyocyanin) to the virulence of *P. aeruginosa* has been demonstrated by several previous studies [72]. *P. aeruginosa* produces a major siderophore (iron-chelating molecule) known as pyoverdine necessary for infection in several different disease models [73]. Pyoverdin is secreted in an apo-form and imported as ferric complexes of iron-binding proteins such as lactoferrin, transferrin and ferritin allowing *P. aeruginosa* to thrive in the iron-poor environment that is the host. Moreover, pyoverdine-dependent iron transport (iron as a cofactor for many metabolic enzymes) is also crucial for biofilm formation [74], highlighting the importance of monitoring the production of these siderophore to study *P. aeruginosa* pathogenesis. Pyocyanin is one of the numerous exotoxins produced and secreted by *P. aeruginosa*. It is a secondary metabolite with the ability to oxidize and reduce other molecules and is able to target a wide range of cellular components and pathways [75].

**Table 3:** Minimum inhibitory concentration (MIC), minimum bactericidal concentration (MBC) and minimum biofilm eradication concentration (MBEC) of the crude organic extract of marine isolate KS8 (mg/mL) against a range of human pathogens associated to indwelling medical device infections.

	PAO1	*E. coli* ATCC 11303	*P. mir.* ATCC 7002	*S. aureus* ATCC 29213	*S. aureus* NCTC 12981	*S. aureus* ATCC 43300	MRSA ATCC 33593	*S. epi.* NCTC 13360	MRSE NCTC 11964
MIC (mg/mL)	2	0.5	1	>4	>4	>4	>4	>4	>4
MBC (mg/mL)	>4	4	2	>4	>4	>4	>4	>4	>4
MBEC (mg/mL)	>4	>4	>4	>4	>4	>4	n.a.	>4	>4

**Figure 8:**
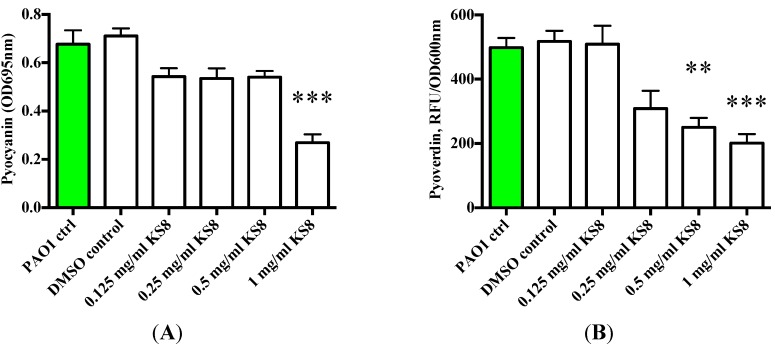
(**A**) Production of Pyocyanain (OD695 nm) by *P. aeruginosa* (PAO1) 48 h biofilms and planktonic cultures following 24 h treatment with KS8 crude organic extract. (**B**) Production of the QS-controlled siderophore Pyoverdine (Excitation 400 nm emission 450 nm/OD600 nm)) by *P. aeruginosa* (PAO1) 48 h biofilms and planktonic cultures following 24 h treatment with KS8 crude organic extract. Differences in mean absorbance were compared to the untreated control at each time-point and considered significant when *p* < 0.05 (* *p* < 0.05, ** *p* < 0.01, *** *p* < 0.001) according to the non-parametric Kruskal Wallis test with Dunn’s Multiple Comparison Test.

To understand whether the putative QSI activity of the crude extracts could affect the QS-dependent synthesis of virulence factors and exoproducts, the production of pyoverdin and pyocyanin by biofilms and their planktonic cultures challenged with KS8 supernatants was measured. The crude extract of isolate KS8 significantly decreased pyocyanin production at the highest concentration tested (1 mg/mL) (*** *p* < 0.001) (Figure 8A). The crude extract of isolate KS8 also significantly decreased the production of pyoverdin at the highest concentration tested (1 mg/mL) (*** *p* < 0.001) (Figure 8B). These results appear to confirm the QSI activity displayed by isolate KS8 on reporter strain *Serratia* sp. ATCC 39006 suggesting the presence of molecules capable of interfering with the C4-HSL pathway of *P. aeruginosa* responsible for regulating pyocyanin and pyoverdin biosythesis.

**Figure 9:**
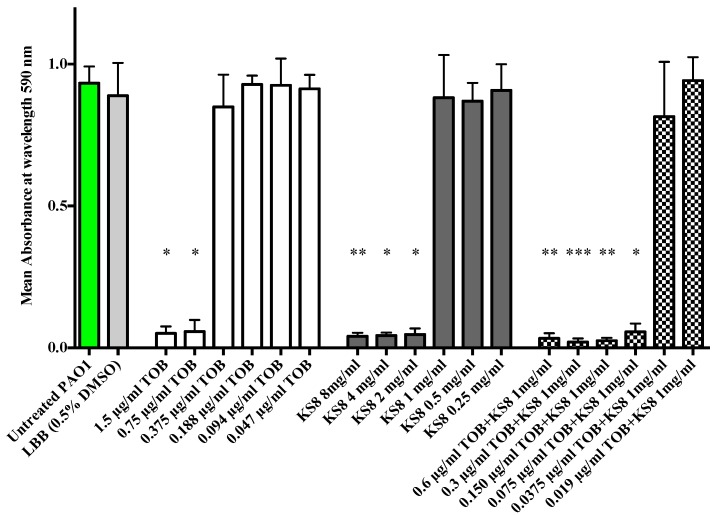
Enhanced antimicrobial activity of tobramycin when used in combination with KS8 organic extract against planktonic cultures of *P. aeruginosa* PAO1. The MIC value of tobramycin against PAO1 was determined to be 0.75 µg/mL. The MIC value of KS8 extract was determined to be 2.0 mg/mL. The MIC value of tobramycin used in combination with KS8 organic extract (1 mg/mL) was 0.075 µg/mL, a ten-fold decrease when compared to the MIC of tobramycin alone. DMSO in LB broth (0.5% w/v) was used as solvent control. Differences in mean absorbance were compared to the untreated control at each time-point and considered significant when *p* < 0.05 (* *p* < 0.05, ** *p* < 0.01, *** *p* < 0.001) according to the non-parametric Kruskal Wallis test with Dunn’s Multiple Comparison Test.

When in the biofilm mode of growth, *P. aeruginosa* utilizes QS to control expression of tissue-damaging virulence factors, including proteases and rhamnolipids which function as a protective shield against the innate immune system causing necrosis of polymorphonuclear leucocytes (PMNs) [76]. *P. aeruginosa* biofilms of mutants lacking a functional QS system have shown to be more susceptible to tobramycin* in vitro* supporting the validity of using QSIs in combination to tobramycin to eradicate biofilm-related infections [15]. The synergistic antimicrobial efficacy obtained by combining KS8 crude organic extract and tobramycin produced a 10-fold decrease in the MIC concentration of tobramycin (from 0.75 µg/mL to 0.075 µg/mL) against *P. aeruginosa* PAO1 (Figure 9). Although the enhanced antimicrobial efficacy remains to be tested *in vivo*, the results suggest that an early, combined treatment strategy for chronic infections using putative QSIs produced by KS8 in combination with tobramycin prophylactically or at an early stage of infection could result in an increased clearance of *P. aeruginosa*, making this strain an ideal candidate for the isolation of novel antibiofilm QSI compounds for the attenuation of virulence and increased antimicrobial susceptibility of *P. aeruginosa*.

In this study, a marine *Pseudoalteromonas* sp. isolate was found to produce putative QSIs active against *P. aeruginosa* PAO1. The supernatant of isolate KS8 caused a significant decrease in both biofilm biomass and viable counts during *P. aeruginosa* biofilm formation and eradication. KS8 extract also caused a significant decrease in the production of pyoverdin and pyocyanin in *P. aeruginosa* biofilm cultures treated with KS8 supernatant supporting the role of QSIs in the antibiofilm activities observed. The efficacy of a combinatorial treatment using tobramycin and KS8-derived QSIs against *P. aeruginosa* PAO1 was investigated *in vitro*, further validating the strategy based on combining QSIs with sub-therapeutic concentrations of conventional antimicrobial agents. Isolate KS8 remains an ideal candidate for the purification and characterization of QSIs with clinical relevance.

## 3. Experimental Section

### 3.1. Bacterial Strains Used in This Study

The test pathogens used in this study are *Pseudomonas aeruginosa* (PAO1), *Staphylococcus epidermidis* NCTC 13360, *S. epidermidis* NCTC 11964, *Staphylococcus* *aureus* ATCC 29213, *S. aureus* ATCC 33593, *S. aureus* ATCC 43300, *S. aureus* NCTC 12981, *Escherichia coli* ATCC 11303, and *P. mirabilis* ATCC 7002.

*C. violaceum* ATCC 12472 (cultured in Luria Bertani (LB) broth at 37 °C), *C. violaceum* CV026 (cultured in Luria Bertani (LB) broth at 37 °C and maintained in kanamycin 25 µg/mL) and *Serratia* sp. ATCC 39006 (cultured in LB broth at 26 °C) were used as QSI reporter strains. *B. cereus* NCTC 9945 harboring the *aiiA* lactonase gene was used as QSI positive control. All strains used in this study were stored in LB broth supplemented with 15% glycerol at −80 °C.

### 3.2. Sampling and Isolation of Bacterial Epiphytes

A variety of marine samples including seaweeds and seagrasses, invertebrates, sediment and sea water were collected in the rockpools in the intertidal zone of 6 different sampling sites along the coast of Ireland (Co. Donegal) and Northern Ireland (Co. Down). Specimens were collected at low tide, trying to avoid heavy perturbance of the surrounding sediments and using sterile gloves and a sterile scalpel. Specimens were collected in sterile 50 mL tubes (BD Falcon™ 50 mL conical centrifuge tubes) or small sterile plastic bags containing seawater prior to removal from the rockpool in order to avoid contact with air. Each sample was kept individually in either 50 mL tubes or plastic bags and stored on ice until processing within a few hours from collection. A portion of each sample was immediately used for the isolation of surface-associated bacteria (within 3 h after arrival at the laboratory).

In order to remove any non-specific, loosely attached and planktonic bacteria prior to the isolation of surface associated epibionts, each eukaryotic specimen (or a section thereof) was placed in a 50 mL conical centrifuge tube with 30 mL of sterile sea water (SSW), vortexed vigorously for 30 s and the supernatant discarded. The process was repeated three times. Rinsed specimens were placed in sterile Petri dishes and manipulated using sterile forceps. A sterile cotton swab was used to collect bacterial epiphytes from the samples’ surfaces and was then placed in 30 mL of Marine Broth (MB) in 50 mL conical centrifuge tubes. Inoculated tubes were incubated at 4 °C, room temperature (rt, 30 °C), 37 °C and 55 °C and examined at regular 24 h-intervals for growth. Once growth was evident in the inoculated tubes, serial dilutions to 10^−7^ in MB were prepared and plated on Marine agar (MA), LB agar (LBA), Skim Milk agar (SMA), Low Swarm agar (LSWA) and Blood Agar (BA) plates and incubated at 4 °C, rt, 30 °C, 37 °C and 55 °C for up to three weeks.

All isolation plates were examined daily for the first week and weekly thereafter. Colonies showing different morphologies (color, texture, shape, size) under the different selective conditions were picked and purified by re-streaking as required on the media used for the isolation. Pure bacterial cultures were stored at −80 °C using the Microbank™ long term storage system (Pro-Lab Diagnostics, Neston, Wirral, UK) as per manufacturer’s guidelines, in triplicate. All isolates were further characterized by examining optimal growth temperature (4 °C, rt, 30 °C, 37 °C and 55 °C) and media (MA, LBA, SMA, LSWA and BA), subjecting them to Gram stain, and screening for haemolytic activity on BA, caseinolytic activity on SMA and catalase production using 3% H_2_O_2_ prior to 16S rRNA gene sequencing.

### 3.3. 16S rRNA Gene Sequencing and Bacterial Identification

Identification of isolates was achieved through 16S rRNA gene sequencing. Total DNA was extracted using the GenElute^®^ Bacterial Genomic DNA kit NA2110-1KT (Sigma Aldrich, Poole, Dorset), as per manufacturer’s guidelines. PCR was used to perform the amplification of the bacterial 16S rRNA gene using the 27F forward primer (5′-AGA GTT TGA TCM TGG CTC AG-3′) and the 1492R reverse primer (5′-TAC GGY TAC CTT GTT ACG ACT T-3′) [77]. The concentration of each DNA sample was measured using the Qubit^®^ Fluorometer (Invitrogen, Paisley, UK) and diluted to 0.5 µg/µL. PCR reactions were prepared by adding 2 µL of genomic DNA sample, 5 µL of primer 27F (2.5 mM) and 5 µL of primer 1492R (2.5 mM) to 25 µL of DreamTaq^®^ Master Mix (Fermentas, Roskilde, Denmark) and 13 µL of double-distilled water (DDW) (total reaction volume 50 µL). A negative control containing additional 2 µL of DDW instead of DNA was prepared. The 16S rRNA gene fragments were amplified using the I-Cycler™ thermocycler (BioRad, Hertfordshire, UK) using the following parameters: initial denaturing step at 94 °C for 3 min, 30 cycles of denaturing temperature at 94 °C for 1 min followed by an annealing temperature of 53 °C for 1 min and extension at 72 °C for 1 min, followed by a final single extension step of 72 °C for 10 min. The products from the amplification were then purified using the Pure Link PCR purification kit (Invitrogen, Paisley, UK), according to the manufacturer’s protocol. Gel electrophoresis using a 1% w/v agarose gel (Invitrogen, Paisley, UK) was carried out on both PCR products and purified PCR products in order to determine quality, concentration and verify the size of the amplicons. The concentration of each purified sample was measured using the Qubit^®^ Fluorometer (Invitrogen, Paisley, UK) and diluted to a concentration of 10 ng/µL prior to sequencing. Sequencing was carried out by Eurofins MWG Operon (London, UK) with a Roche GS FLX sequencer using the 27F primer. Several strains were further sequenced using the 1492R primer in order to improve identification. Sequence data were analyzed by comparison with the 16S rRNA gene sequences available in the National Center for Biotechnology Information (NCBI) GenBank database. Isolate identification was performed using the BLASTn server tool.

### 3.4. Antimicrobial Screening Assays

The disc diffusion susceptibility method was performed according to the method described by Bauer *et al.* [78] and used to screen supernatants and the crude organic extracts of isolate KS8 for the presence of antimicrobial compounds against *Escherichia coli* ATCC 11303, *Pseudomonas aeruginosa* PAO1 and *Proteus mirabilis* ATCC 7002. Antimicrobial activity was quantified by modifying the paper disc diffusion procedure described by Assefa *et al.* [79]. The antibiotic susceptibility of isolate KS8 was determined using Oxoid M.I.C.Evaluator™ (M.I.C.E™; Thermo Fisher Scientific, Waltham, MA, USA) [80].

### 3.5. Screening for the Production of QSI

*Chromobacterium violaceum* ATCC 12472 (cultured in LB broth at 37 °C), *C. violaceum* CV026 (cultured at 28 °C in LB broth supplemented with Kanamycin 25 μg/mL), and *Serratia* sp. ATCC 39006 (cultured in LB broth at 26 °C) were used to screen for the presence of QSI activity. Screening for the production of QSIs was carried out according to the protocol described by McLean *et al.* [81]. Marine isolates were streaked on LB agar plates and incubated at 28 °C for 24–48 h. Plates were then overlaid with 10 mL of 0.5% LB agar (47 °C) inoculated with 5 µL of an overnight culture of *C. violaceum* (ATCC 12474) or 5 µL of an overnight culture of *Serratia* sp. (ATCC 39006) or 10 mL of 0.5% LBA 5 µM *N*-(3oxo-hexanoyl)-dl-homoserine lactone (3OC6HSL) (Sigma Aldrich, Poole, Dorset) inoculated with 50 µL of an overnight culture of CV026. Plates were incubated overnight at 37 °C, 28 °C or 26 °C depending on the reporter strain being used. Following overnight incubation, the presence of an opaque halo (growth of reporter strain with pigment inhibition) surrounding the isolate being screened was considered indicative of putative QSI activity. Growth inhibition of the indicator culture (the presence of a clear halo surrounding the isolate being screened) was considered indicative of possible antimicrobial activity.

### 3.6. Preparation of Culture Supernatants and “Conditioned Media”

Microbank™ long-term preserver beads (Pro-Lab Diagnostics, Neston, Wirral, UK) were used to streak LBA plates with the marine isolates to be tested. Single colonies were picked and used to inoculate 100 mL of LB broth in glass flasks. Flasks were incubated at rt, 28 °C or 37 °C at 100 rpm in a gyrorotary incubator and left for the required amount of time (1, 3, 6, 9 or 10 days). The cultures were then centrifuged at 4000 rpm for 30 min. 15 mL aliquots of the cell-free cultures were then transferred to a 20 mL syringe and sterilised using 0.45 µm and 0.20 µm pore size filters in series (Millipore, Carrigtwohill, Ireland). A volume of 30–45 mL of each supernatant was collected in sterile tubes and stored at −20 °C.

In order to verify the impact of using nutrient-depleted supernatants to study antibiofilm activity on different test strains, conditioned media (CM), consisting of the sterile supernatant of the different test strains (*E. coli*,* P. mirabilis* and* P. aeruginosa*) grown in LB broth for different amounts of time was prepared as described above and used as a control where appropriate.

### 3.7. Biofilm Formation Assay

To measure the biomass produced (biofilm formation) by *E. coli*,* P. mirabilis* and *P. aeruginosa* in the presence of the sterile supernatants of the marine isolates, test biofilms were grown in 96-well microtitre plates using the method described by Stepanovic *et al.* [82], with slight modifications. Overnight inoculums of test strains were diluted to a 0.5 McFarland standard (approx. OD 0.3, 550 nm), using quarter strength Ringer’s solution (QSRS) corresponding to a total planktonic viable count of approximately 1.5 × 10^8^ colony forming units per milliliter (CFU/mL). The inocula were further diluted to 3 × 10^6^ CFU/mL (verified by Miles and Misra viable count [83]). 100 µL of the inoculum of each test pathogen was placed in columns 2–12 of a 96-well microtitre plate. 200 µL of LB broth were placed in column 1 as a sterility control. 100 µL of filter sterilised supernatants of the marine bacterial isolates were placed in columns 4–12 of the 96-well microtitre plates. 100 µL of LB broth were added to column two as an untreated control. In order to verify the effect of nutrient depletion on biofilm formation, 100 µL of conditioned media (CM) was added to column three as an additional control. Plates were incubated and biofilms allowed to grow for 24 h at 37 °C and 95% relative humidity and 150 rpm in a gyrorotary incubator. Following exposure to spent supernatants for 24 h, the adherent biomass of each well was washed three times with 280 µL of PBS solution to remove planktonic bacteria. The biofilms were then fixed by adding 250 µL of MeOH and incubating at room temperature for 30 min. The methanol was then discarded and the plates allowed to air dry for 10 min under a laminar air hood. Being careful to avoid staining the thick air liquid interface, 180 µL of 0.1% (w/v) crystal violet solution in 70% ethanol were added to each well and left for 5 min at rt. The crystal violet solution was then discarded and the plates rinsed 3 times with 300 µL of 0.9% saline solution making sure that no excess unbound crystal violet had remained. The plates were then allowed to air-dry for 15 min. Finally, the stain bound to the biofilm matrix was re-solubilised with a mixture of 80% EtOH and 20% acetone. The mean absorbance of the biofilm biomass was measured at 590 nm using a Tecan Sunrise^®^ plate reader (Männedorf, Switzerland). Each crystal violet assay was run in triplicate, with a minimum of 6 replicates per assay.

### 3.8. Biofilm Removal Assay

The Calgary Biofilm Device (CBD) (available as the MBEC AssayTM for Physiology & Genetics (P&G), Innovotech Inc., Edmonton, Alberta, Canada) was used to evaluate the effect of filter-sterilised supernatants on the biomass of pre-established 24 h biofilms of test pathogens. The wells of the CBD were inoculated with 200 µL of a 3 × 10^6^ CFU/mL inoculum of test strains prepared as previously described. LB broth was included in each assay as a sterility control. The MBEC assay plates (Innovotech Inc., AB, Canada) were transferred to a gyrorotary incubator (37 °C, 95% relative humidity) for 24 h to allow growth of test biofilms. A separate challenge plate was inoculated with 200 µL of isolates’ supernatants diluted in LB broth (50:50 v/v). LB broth was included in each assay plate as a sterility control and to provide an untreated control. The effect of nutrient depletion was determined growing each test strain in a mixture of conditioned media (CM) and LB broth (50:50 v/v). Following 24 h of incubation, the lid of the CBD was rinsed three times by immersing the pegs in three separate 96-well plates containing 250 µL of QSRS. The lid with was then placed on the corresponding challenge plates and incubated at 37 °C and 95% relative humidity and 150 rpm in a gyrorotary incubator for 24 h. Following exposure of test biofilms to the supernatants for 24 h, biofilms were rinsed with QSRS, fixed with methanol, air-dried and stained by immerging the lid in a 96-well plate containing 180 µL of 0.1% crystal violet solution per well. Finally, the stain bound to the biofilm matrix was re-solubilized in a 96-well plate containing 200 µL of a mixture of ethyl acetate EtOH and acetone (4:1). The mean absorbance of the biofilm biomass was measured at 590 nm using a Tecan Sunrise^®^ plate reader (Männedorf, Switzerland). Each assay was run in triplicate, with a minimum of 6 replicates per assay.

### 3.9. Preparation of Crude Culture Extracts

A volume of 1 mL of an overnight culture of marine isolate KS8 was used to inoculate individual glass flasks containing 1 L of LB broth. Flasks were incubated at rt, 28 or 37 °C with constant shaking at 150 rpm in a gyrorotary incubator. Following incubation for 1, 3, 6, 8 or 10 days, the 1 L cultures were extracted three times with 1 L of EtOAc at rt. Each extraction was performed in 2.5 L bottles placed on a horizontal shaker at 200 rpm for 1 h. The resulting two phases were separated and recovered using a separating funnel, and the extraction repeated. The combined organic phases of each supernatant (3 L) were dried using a rotary evaporator Rotavapor (R-114, Büchi, UK) at a temperature of no more than 37 °C. Dried extracts were stored at −20 °C.

### 3.10. QSI Disc Diffusion Assay

The QSI disc diffusion assays on test strains were performed using sterile supernatants and the organic extracts. Dried organic extracts were re-solubilized in EtOAc to yield solutions of 10 mg/mL. The re-solubilized solutions were pipetted onto sterile paper disks 6 mm in diameter (Whatman, UK) by transferring a maximum of 10 µL volumes at a time to achieve the desired test concentrations. Air-drying was allowed between multiple loadings. A disc loaded with 100 µL of solvent (EtOAc unless otherwise specified) and allowed to air dry was also included as a negative control. The LBA plates were then overlaid with 10 mL of 0.5% LBA inoculated with 5 µL of an overnight culture of *C. violaceum* (ATCC 12474) or 10 mL of 0.5% LBA 5 µM *N*-(3oxo-hexanoyl)-dl-homoserine lactone (3OC6HSL) (Sigma Aldrich, Poole, Dorset) inoculated with 50 µL of an overnight culture of CV026. Plates were incubated overnight at 37 °C for 24 h before being examined for the presence of opaque halos indicating QSI inhibition or clear halos indicating antimicrobial activity.

### 3.11. MIC/MBC Determination

Broth microdilution tests were carried out based on the protocol described in the NCCLS guidelines [84], with slight modifications. Crude organic extracts were re-solubilized in DMSO and a working solution of each extract to be tested was prepared in LB broth (0.5% DMSO) and sterilized using a 0.22 µm filter. From this stock solution, serial two-fold dilutions in LB broth were carried out in 96-well microtitre plates over the concentration range 8–0.0078 mg/mL. A control using LB broth with 0.5% DMSO was also included in each broth microdilution assay.

### 3.12. Screening for Antibiofilm Activity

To test the effect of the filter-sterile supernatants or the crude organic extracts of the marine isolates on the viability of both biofilm and planktonic cultures during both biofilm formation and biofilm eradication (of mature biofilms) of test strains *P. aeruginosa* PAO1, *E. coli* ATCC 11303 and *P. mirabilis* ATCC 7002, the original MBEC protocol [85] was modified in order to generate three different sets of data including viable biofilm counts, MIC and MBEC values. Test strains were grown overnight in LB broth at 37 °C and then subsequently diluted to obtain a culture of 0.3 OD at 550 nm and further adjusted to provide a final inoculum density of ~10^7^ CFU/mL (confirmed by viable counts). The biofilms of each test organism were grown in the Calgary Biofilm Device (Innovotech Inc., Edmonton, AL, Canada).

#### 3.12.1. Anti-Adherence Assay-Viable Counts

An MBEC plate was inoculated as follows: column 1 containing 200 µL of LB broth (blank), column 2 containing 100 µL of LB broth and 100 µL of test inoculum (negative control), column 3 containing 100 µL of conditioned media when testing supernatants or 100 µL of media with solvent control (0.5% DMSO) when testing crude extracts and 100 µL of test inoculum, and columns 4–12 containing 100 µL of the marine isolate’s supernatant or crude extract in LB broth together with 100 µL of test strain inoculum. Plates were incubated and biofilms allowed to grow for 24 h at 37 °C and 95% relative humidity and 100 rpm in a gyrorotary incubator.

Following 24 h of incubation the plate was removed from the incubator and the lid of the 96 well plate was rinsed to remove planktonic bacteria by placing it into two fresh plates containing 250 µL of sterile saline solution (0.9%) per well for 1 min. After rinsing the biofilms, four pegs were broken off from each of the test columns using sterile pliers and placed in the wells of the top row of a fresh 96 well plate containing 200 µL of sterile saline (0.9%) solution per well. The plate was then sonicated for 20 min, an interval of time previously optimized and shown not to reduce *P. aeruginosa* PAO1, *E. coli* ATCC 11303 and *P. mirabilis* ATCC 7002 viability, using a Branson 3510 ultrasonic cleaner (40 KHz, 130–335 W) (Branson, Danbury, CT, USA). Following sonication, the pegs were discarded and each sample was serially diluted to 10^−11^. Each sample dilution was then plated using the Miles and Misra technique [83] and incubated overnight at 37 °C. The growth of the test strains’ biofilms on the pegs was expressed as biofilm forming units per peg (BFU/peg).

#### 3.12.2. Screening for Antibiofilm Compounds with Biofilm Eradication Activity (MBEC)-Viable Counts

The MBEC assay was conducted using KS8 organic extract prepared as for the MIC tests, as previously described. The wells of the CBD were inoculated with 200 µL of a 3 × 10^6^ CFU/mL inoculum of test strains prepared as previously described. The MBEC assay plates were transferred to a gyrorotary incubator (37 °C, 95% relative humidity) for 24 h to allow growth of test biofilms. Biofilm viable counts (BFU/peg) at 24 h were measured. Following the 24 h growth period, the peg lid of the MBEC assay plate was transferred to a rinse plate and each peg gently rinsed three times by immersion in wells containing 300 µL sterile PBS.

After rinsing, the lid was transferred to a challenge plate containing serial doubling dilutions of KS8 extract over the concentration range of 4–0.0156 mg/mL (final volume 200 µL). LB broth and DMSO in LB broth (0.5% w/v) were used as negative and solvent control. The MBEC assay plates were transferred to a gyrorotary incubator (37 °C, 95% relative humidity) for 24 h. Following exposure of the biofilms for 24 h the lid with the pegs was removed from the challenge plate and rinsed three times in 300 µL of sterile PBS. After rinsing, four pegs were broken off from each of the test columns and used to determine biofilm viable counts. The lid with the remaining pegs was transferred to a “recovery” plate containing LB broth. Biofilms were sonicated for 5 min and the peg lid discarded. The recovery plate was incubated overnight and checked visually after 24 h for turbidity. An MBEC value was assigned as the lowest concentration at which no growth was observed after 24 h incubation.

### 3.13. Measurement of Siderophore (Pyoverdin) and Exoproduct (Pyocyanin) Production

Pyoverdin production by *P. aeruginosa* PAO1 biofilms was measured according to the protocol described by Romanowski *et al.* [86], with slight modifications. An MBEC device was inoculated with PAO1 (inoculum density of ~10^7 ^CFU/mL, as confirmed by viable count, 200 µL/well) and biofilms were allowed to develop for 24 h at 37 °C in LB broth. Following 24 h incubation, the MBEC lid with the 24 h biofilms was rinsed three times by placing it into three separate 96-well plates each with 250 µL of PBS in each well. Following rinsing, the lid was transferred to black, clear bottom 96-well plates (Corning Incorporated, Corning, NY, USA) containing the treatment to be applied. Following 24 h incubation with extracts, pyoverdin production was measured by fluorescence (ex 400 nm, em 460 nm) using a BMG Fluostar Optima Fluorescence Plate Reader (BMG Labtech Ltd., Aylesbury, UK). The measurement of the Pyocyanin produced by *P. aeruginosa* PAO1 biofilms was performed using the method described by [87], with slight modifications.

### 3.14. Scanning Electron Microscopy (SEM) of Biofilms

Following exposure of pathogenic biofilms to the marine isolates’ supernatants or crude extracts for 24 h in the CBD, three pegs per treatment, randomly chosen, were removed from the lid of the device and rinsed with 250 μL of 0.9% saline for one minute to remove planktonic and loosely adherent bacteria. The pegs were fixed in separate 1.5 mL eppendorf tubes containing 1 mL of 3% glutaraldehyde in 0.1 M cacodylic acid (pH 7.2) overnight, at 4 °C. Following fixation, pegs were washed four times for 30 min with 250 μL of 0.1 M cacodylate buffer at 4 °C. Immediately after the wash, samples were dehydrated at rt in five steps by placing the pegs for 30 min in 250 μL of 50%, 70% and 90% and then twice for 30 min in 100% ethanol. The pegs were then transferred to a microtitre plate containing 200 µL of hexamethyldisilazane (HMDS) and left to dry for 24 h in a fume cupboard. The specimens were mounted on aluminum stubs, gold sputter coated, and examined using the field emission scanning electron microscope FE-SEM (JEOL JSM-6500F FE-SEM, JEOL Ltd., Tokyo, Japan) or FE-SEM (JEOL JSM-840 SEM, JEOL Ltd., Tokyo, Japan) in which case observations were performed at 15 kV and images captured onto Ilford black and white film.

### 3.15. Combinatorial Treatment of PAO1 Biofilms

To study whether the putative QSI bioactive(s) produced by isolate KS8 could improve the antimicrobial efficacy of tobramycin against planktonic populations of *P. aeruginosa* PAO1, the crude organic extract was tested for its MIC value in combination to tobramycin. To determine the MIC of tobramycin alone, a broth microdilution assay was carried out based on the protocol described in the NCCLS guidelines [84]. In accordance with Christensen* et al*. [76], the MIC of tobramycin against *P. aeruginosa* PAO1 was found to be 0.75 mg/L.

To determine the MIC of tobramycin for PAO1 when used in combination with a sub-MIC (1 mg/mL) concentration of KS8 crude organic extract, the crude organic extract was re-solubilized in DMSO and a solution (4 mg/mL) of the extract to be tested was prepared in LB broth (2% DMSO) and sterilized using a 0.22 µm filter. 50 µL of the working solution was pipetted in columns 4–12 of a microtitre plate. 50 µL of tobramycin in LB broth over the concentration range of 30–0.0003 µg/mL was added to each well of columns 4–12. Finally, 100 µL of PAO1 inoculum were added to all wells of columns 4–12. A sterility control (200 µL of LB broth) was placed in the wells of column one. An untreated control (100 µL of LB broth + 100 µL of PAO1 inoculum) was placed in the wells of column two. A solvent control (100 µL of LB broth with 1% DMSO + 100 µL of PAO1 inoculum) was added to the wells of column three. As a result, the initial working solution (4 mg/mL 4% DMSO) of KS8 crude extract and the concentration range of tobramycin will both have been diluted 4-fold to 1 mg/mL and 7.5–0.00075 µg/mL respectively. Plates were placed in an incubator at 37 °C for 24 h prior to being read at 590 nm OD. MIC determination was repeated using doubling dilutions of tobramycin and KS8 extract.

### 3.16. Statistical Analysis

Statistical analysis was performed using GraphPad Prism™ 5.01 (GraphPad Software Inc., San Diego, CA, USA). In the experiments to screen isolate’s supernatants for antibiofilm activity, viable counts of biofilms challenged with each supernatant were compared to untreated biofilms of test organisms, unless otherwise stated. The non-parametric Mann Whitney U-Test was used to compare the effect of each single challenge to the untreated control. The non-parametric Kruskal Wallis test, with a Dunn’s Multiple Comparison Test, was used for comparisons between more than two groups and to identify individual differences. All colony count data underwent log_10_ transformation before statistical analysis to normalize the data. Differences compared to controls were considered significant when *p* < 0.05 (* *p* < 0.05; ** *p* < 0.01; *** *p* < 0.001).

## 4. Conclusions

The Gram-negative opportunistic human pathogen *Pseudomonas aeruginosa* is a leading causative organism of multi-drug resistant nosocomial infections [88]. In this study, a panel of marine bacteria isolated from the surface of different eukaryotic organisms collected in the rock pools of the intertidal zone of the coast of Ireland and Northern Ireland were screened for QSI and antibiofilm activity against *P. aeruginosa* PAO1. Isolate KS8, identified as a *Pseudoalteromonas* sp., was found to display strong QSI activity against pigment-based reporter strains *C. violaceum* ATCC 12472, CV026 and *Serratia* sp. ATCC 39006 and to cause a significant decrease in biomass and viable counts in *P. aeruginosa* PAO1 biofilms. The crude organic extract of KS8 inhibited the production of pyocyanin and pyoverdin in *P. aeruginosa* PAO1 further supporting the involvement of QSIs in the antibiofilm effects observed. To conclude, KS8 organic extract and tobramycin were used in a combinatorial approach to determine whether the presence of putative QSIs could enhance the antimicrobial activity of this antibiotic. The MIC of tobramycin used in combination with KS8 extract was 0.075 µg/mL, ten times lower than tobramycin alone, further supporting the validity of strategies relying on the use of QSIs to reduce the therapeutic dose of antibiotics.

## Supplementary Files

Supplementary File 1
