# Supplementary material for: Marine-Derived Quorum-Sensing Inhibitory Activities Enhance the Antibacterial Efficacy of Tobramycin against Pseudomonas aeruginosa
Source: Mar Drugs. 2014 Dec 24;13(1):1–28. doi: 10.3390/md13010001 (PMC4306922; doi:10.3390/md13010001)
Supplement: Supplementary File 1 [file marinedrugs-13-00001-s001.pdf]

Supplementary Information

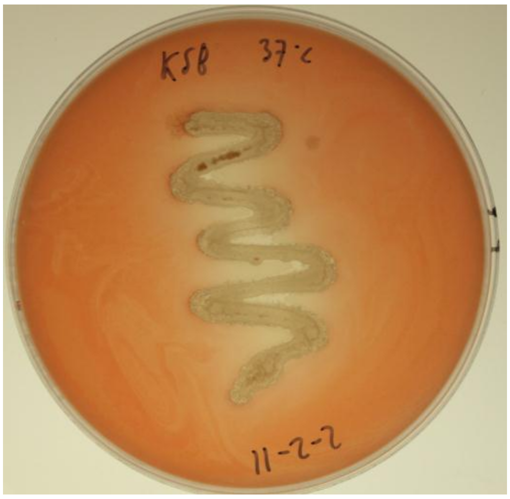

Figure S1. Screening KS8 for QSI activity using reporter strain *Serratia* sp. ATCC 39006. Inhibition of pigment production in the proximity of KS8 is indicative of positive QSI activity.

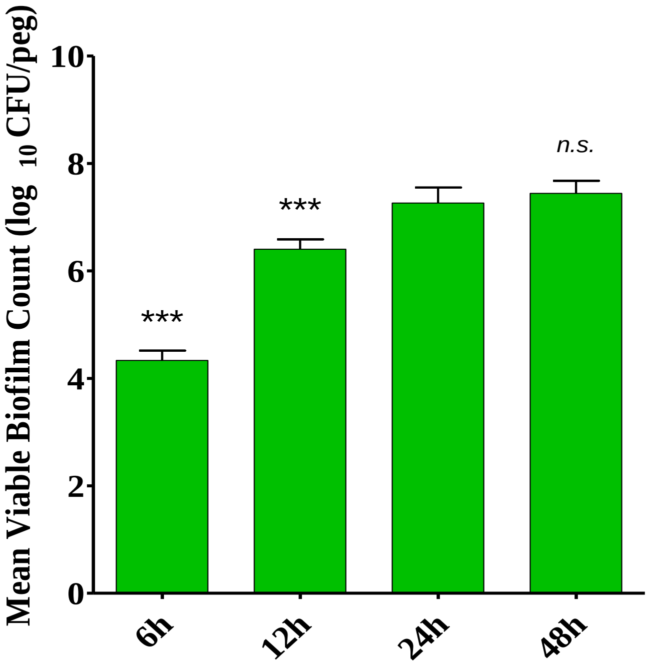

Figure S2. Mean Biofilm Counts of *P. aeruginosa* PAO1 over 48 h. Biofilms were grown on the Calgary Biofilm Device for 48 h in LBB at 37 ℃ and 150 rpm. Each value is expressed as the mean and standard deviation of six replicates. Differences in counts compared to the 24h time point were considered significant when $p < 0.05$ (* $p < 0.05$, ** $p < 0.01$, *** $p < 0.001$) according to the non parametric Kruskal with Dunn's Multiple Comparison Test. (n.s. = not significant).

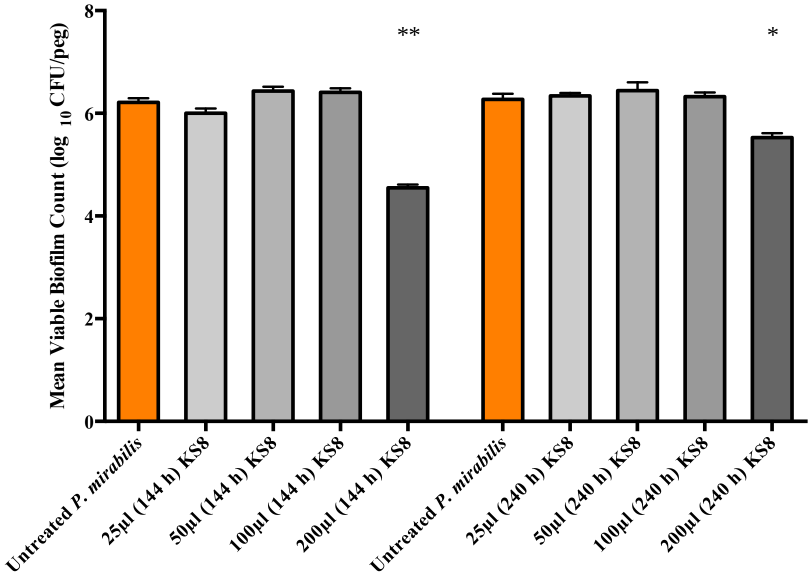

Figure S3. Prevention of biofilm formation by isolate KS8 supernatant. *P. mirabilis* ATCC7002 mean biofilm counts following 24 h exposure to sterile 6-day and 10-day supernatant of isolate KS8. *P. mirabilis* biofilms were grown on the Calgary Biofilm Device for 24 h in LBB at 37 °C and 150 rpm.

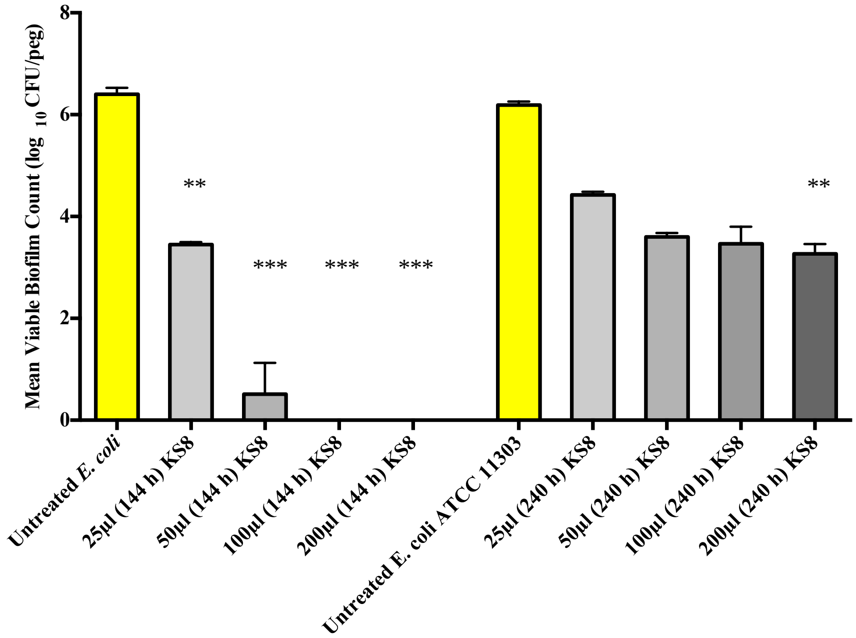

Figure S4. Prevention of biofilm formation by isolate KS8 supernatant. *E. coli* (ATCC11303) mean biofilm counts following 24 h exposure to sterile 6-day and 10-day supernatant of isolate KS8. *E. coli* (ATCC11303) biofilms were grown on the Calgary Biofilm Device for 24 h in LBB at 37 °C and 150 rpm.

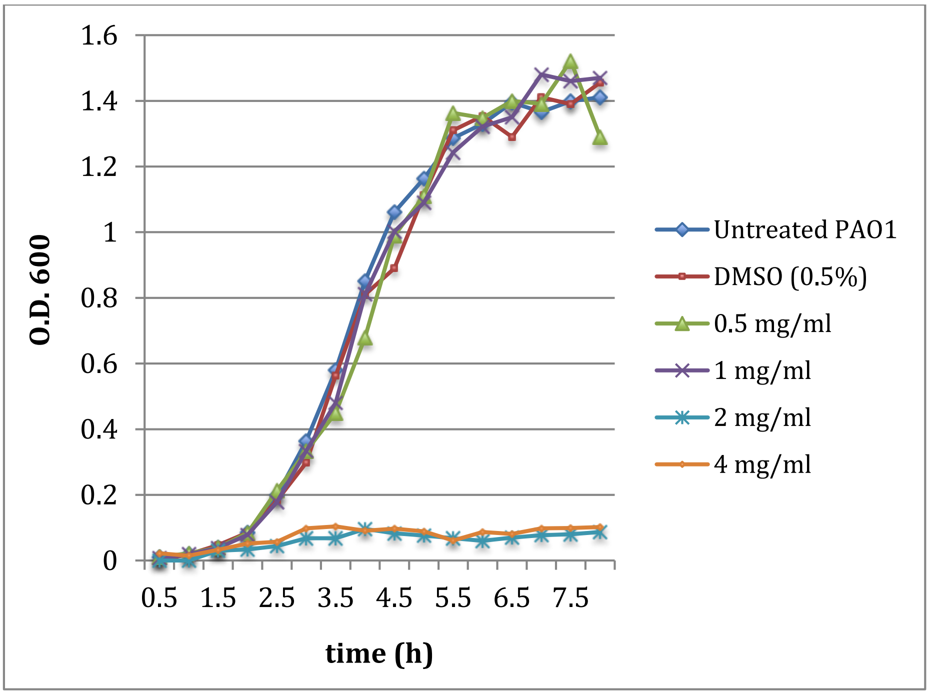

Figure S5. Planktonic growth curves (O.D. 600) of *P. aeruginosa* PAO1 in LB broth (37 °C) in the presence of KS8 crude organic extract. Mean absorbance was measured at 600 nm using an automated Tecan Sunrise® plate reader (Männedorf, Switzerland). Growth inhibitory activity is apparent at the higher concentrations tested (2 and 4 mg/mL).

© 2014 by the authors; licensee MDPI, Basel, Switzerland. This article is an open access article distributed under the terms and conditions of the Creative Commons Attribution license (http://creativecommons.org/licenses/by/4.0/).
